# Objective and Perceived Traffic Safety for Children: A Systematic Literature Review of Traffic and Built Environment Characteristics Related to Safe Travel

**DOI:** 10.3390/ijerph19052641

**Published:** 2022-02-24

**Authors:** Yasser Amiour, E. O. D. Waygood, Pauline E. W. van den Berg

**Affiliations:** 1 Department of Civil, Geological, and Mining Engineering, Polytechnique Montréal, Montreal, QC H3T 1J4, Canada; owen.waygood@polymtl.ca; 2 Department of the Built Environment, Eindhoven University of Technology, 5600 MB Eindhoven, The Netherlands; p.e.w.v.d.berg@tue.nl

**Keywords:** injury, perception of safety, children, active transportation, traffic, road design

## Abstract

The literature on children’s active transportation has shown the influence of the built environment characteristics on walking and crashes. Various reviews have examined those two questions. One influence on walking is the perception of traffic safety. However, it is not clear how, or even if, the built environment affects such perceptions. This research aims to understand which traffic and built environment characteristics influence objective and subjective/perceived traffic safety for children based on the analysis of previous studies in the field. Two types of research were used: the first examines the association between traffic and built environment characteristics and child pedestrian and/or cyclist collisions/injuries; the second relates to the perception of safety by parents and children for active transportation and, where studied, its relationship with built environment characteristics. A systematic review was conducted using five electronic databases. The total number of articles retrieved was reduced to 38 following the eligibility criteria and quality assessment, where 25 articles relate to injuries among children and 13 articles pertain to perception of safety. The results showed that high traffic volume and high vehicle speed are the main reasons children and parents feel unsafe when children use active travel, which matches the main findings on objective safety. Few articles on perception of safety related to the objective built environment were found. However, consistent findings exist. The presence of sidewalk was related to the safety of children. The presence of a crossing guard was positively related to perceived safety but was associated with higher rates of injuries among children. Intersection density was related to unsafe perceptions but was not statistically associated with objective traffic safety. Additionally, population density was found to be positively related to injuries among children, but not to perception of safety. The results help policy strategy to enhance the safety of children when using active transport modes.

## 1. Introduction

Children need to be able to safely travel in the environment where they live whether it is to go to school, play with friends, or engage in other activities. Over the past decade, considerable research has been focused on children’s active transportation to school and how it relates to physical activity [1,2,3,4,5,6]. Related to that, children’s independent mobility continues to be an important topic [7] as children’s independent mobility (CIM) could also increase children’s well-being [8]. CIM is described as: “Children’s freedom to travel around in their neighborhood or city without an adult or parental supervision” [9]. However, a key component of CIM is both perceived and objective traffic safety [10].

Parents are one of the determinants for children’s independent mobility by making decisions on whether or not to let their children walk or bike to school or to other destinations [11]. Parents judge traffic, which they do not have control over, but they also train and socialize their children to use different modes [12]. As such, a parent’s assessment of a child’s skills is also important. Parents feel that long distance and the danger of traffic are key barriers to walking and cycling to school [13,14].

Previous studies focused on the correlation between the built environment, urban neighborhood and the likelihood of walking to school [5,15,16,17]. However, parents’ perception of safety plays a role as well, so we need to consider how this perception is formed. One part is likely linked to the built environment and traffic conditions where people live, but also at the destination.

For children to travel actively and independently, the environment must be safe, and it is important that parents perceive it as safe. Enhancing the safety of children when using active transportation could lead to a decrease in injuries among children and encourage more active and independent travel for children.

A concept of built environment and traffic safety was presented by Ewing and Dumbaugh [18]. The built environment which contains roadway designs and development patterns can affect traffic safety by traffic volume, speed, and conflicts. That research, however, did not directly consider children who must be considered as a distinct group separate from adults due to various factors including limited height (being hidden from drivers by parked vehicles for example), less experience in such contexts, and cognitive development.

A few reviews have examined the influence of the built environment on traffic collisions involving children. Rothman et al. [19] considered the influence of the built environment on both collision risk and the likelihood of walking. Road features such as traffic calming were highlighted as a means of both reducing injury incidence and increasing walking. As well, traffic levels, pedestrian density, road density, crossing major roads, and mixed land use were all associated with increased rates of injury incidence. More recently, Cloutier et al. [20] completed a review from the perspective of a Safe System approach. They highlighted three key influences: the built environment, vehicles, and drivers. Within the built environment, one could include safe speeds linked to both posted speed and street design. As well, children in more socially deprived areas were more likely to be involved in collisions which the authors linked to higher traffic passing through such areas and a higher likelihood of children walking. However, the first [19] was completed in 2014 and the second’s focus is not solely on the built environment and thus does not often go into detail on what was studied and the results. Neither of these reviews examines whether there is an influence of the built environment on parents’ perception of traffic safety.

Two concepts of traffic safety can be considered. One relates to instances of danger or harm, such as near misses or crashes. The other relates to the individual evaluation of safety, or perceived traffic safety. In this paper, the terms objective safety and perceived safety were used such as described in several studies [21,22,23]. Objective traffic safety, also referred to as “real traffic safety” [24], pertains to the number or risk of collisions and any resulting fatalities or injuries caused by road traffic. Perceived traffic safety on the other hand, is the perception of safety or risk caused by road traffic [21,22,24]. In the case of children, perceived traffic safety pertains to parents’ and children’s perception of safety.

Many research papers have examined the ability of children to perceive danger [25,26,27,28,29] or children’s behaviors related to traffic safety [30,31,32,33]. Such studies aim to examine children’s capacity to understand traffic danger and resulting behavior such as when to cross. Such studies are important to adjust traffic so that children may cross safely as is the approach in child safety leading countries such as Sweden [34]. However, this study looks at parents’ and children’s perception of safety related to general traffic conditions (i.e., their impression of traffic volumes, speed, and driver behavior) as it relates to whether or not children would be allowed to travel actively and independently.

McMillan [11] showed the importance of perception of safety of traffic and neighborhood to parental decision making for children’s active and independent travel. The urban form could indirectly affect parental decision making by mediating factors such as neighborhood and traffic safety including real safety (e.g., collision and injuries) and perceived safety (e.g., parent’s perception). In this framework, parents are a key determinant of children’s travel to school—they judge the neighborhood environment and then decide on how their children will travel to school. Mitra and Manaugh [10] developed a social-ecological model of children’s independent mobility (CIM), in which the perception of traffic safety could play a role.

Previous models mentioned above, McMillan [11], Sirard and Slater [35], and Mitra and Manaugh [10], showed the importance of parents’ perception of safety of traffic and neighborhood for children’s active and independent travel. Previous research [36,37,38,39], also showed the influence of built environment and traffic on objective traffic safety (collisions involving children). However, the relationship between the influence of the built environment (and traffic) on objective traffic safety and subjective/perceived traffic is not well clarified and could pose a problem to manage the current situation well. For example, imagine that a specific road design characteristic such as one-way streets increases the number of injuries involving children but suppose that parents believe that one-way streets are safe for their children to walk or cycle, that would be a problem because there is an inverse correlation between objective (collisions involving children) and subjective/perceived traffic safety that would possibly increase children’s travel in a dangerous environment. As such, one of our main objectives is to see where there is agreement and disagreement between objective and subjective measures of traffic safety children while conducting active travel (primarily walking or cycling in studies).

This systematic review aims to understand which traffic and built environment characteristics could influence collisions involving children and perception of safety based on previous related studies, and what relationship exists between objective and perceived traffic safety.

## 2. Materials and Methods

This study was conducted using five electronic databases: Web of Science (2000–2020), PubMed (2000–2020), Compendex (2000–2020), ScienceDirect (2000–2020) and ProQuest Dissertations & Theses (2000–2020). Specific keywords cannot all be presented here (as they are too numerous; Appendix A), but followed these general themes: perception of safety, injury, traffic, built environment, social environment, children, and active transportation.

**Figure 1:**
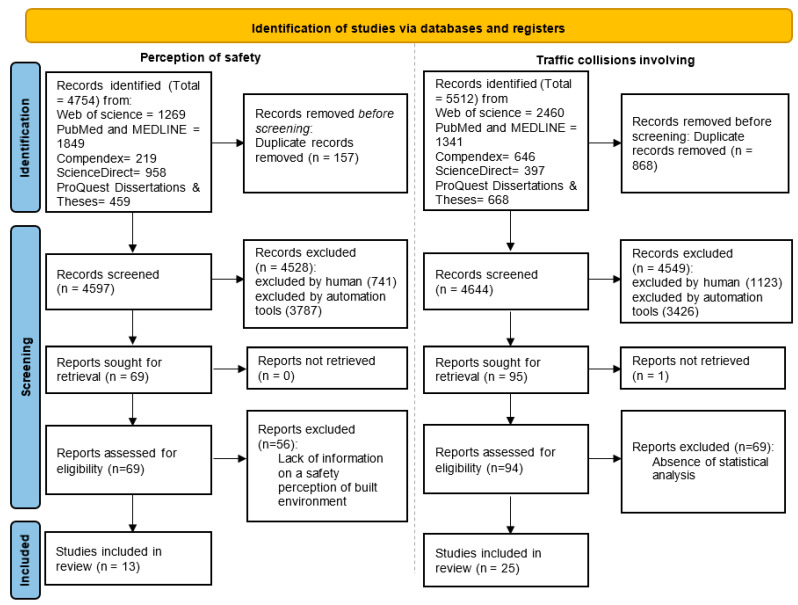
Flow diagram for the systematic review following the PRISMA statement.

Figure 1 shows the flow diagram used to identify the relevant articles of the systematic literature review following the Preferred Reporting Items for Systematic Reviews and Meta-Analyses (PRISMA) statement.

The two types of research conducted in the present literature review aim to identify relevant articles of objective and subjective traffic safety for children. The first type of research was limited to articles that examine the association between child pedestrians’ or child cyclists’ collisions and measures related to traffic and the built environment. The second type of research considered was associated with subjective measures including perception of safety for children’s active travel and its relationship with traffic and built environment measures. Both parents’ and children’s perceptions of safety were considered.

The total number of studies retrieved was reduced to 38 studies following eligibility criteria and quality assessment. Following that step, 25 studies for collisions/injuries involving child pedestrian and cyclists were retained and 13 studies contain perceptions of traffic safety. Among the 13 studies selected for perception of safety, there were only 5 studies that examined the statistical relationship between objective built environment and perception of safety for children. The eight articles (four quantitative and four qualitative studies) contain the parents’ and children’s answers to questions related to traffic safety when using active transportation without examining the statistical relationship with the objective built environment characteristics.

Checklists were used to address the risk of bias in the included studies. First, a librarian in Polytechnique Montreal helped identify and develop a research strategy used in each database. The principal reviewer (Y.A.) examined the articles with the help of some tools (e.g., EndNote) provided in the library of Polytechnique Montreal. Records were also screened by a second reviewer (E.W.) to address the risk of bias.

### 2.1. Selection Criteria

The initial number of studies presented in Figure 1 was reduced to 38 studies retained for analysis. All duplicate studies were removed in the first step before the screening. The second step was to exclude document types such as literature reviews, conference abstracts, book reviews, or encyclopedia entries. Articles that were not related to subject and articles of other disciplines (e.g., medical purpose) were excluded based on information provided in databases.

The next step was to screen the articles. First, we screened articles based on their abstract. We only included articles that present a relationship between these following fields: objective traffic safety (e.g., collision and injuries) and/or perception of safety when using active travel modes (walking or/and cycling) with built environment and/or traffic. Articles that present a relationship between traffic safety and physical activity or obesity were excluded. Only articles related to active travel safety were retained. Children were limited to aged 18 years old or younger, and samples that did not contain school-aged children were excluded.

In the final screening, we excluded articles based on the full text. For collisions/injuries involving children, statistical analyses using various methods (e.g., multivariate analysis) were considered to examine the relationship between collisions/injuries involving children and built environment and/or traffic. For articles on perception of safety, both qualitative and quantitative studies were considered. The first are often based on parents’ responses to interviews or focus groups, while the second often used results from surveys that applied a Likert scale/Point scale to measure perceptions. Some studies examined the association between perception of safety and objective measures of the built environment and traffic. Finally, articles that examine the relationship between objective built environment and either perception of safety or traffic collisions involving children are included and regrouped.

### 2.2. Analysis Procedure

For objective traffic safety (traffic collisions involving children), the outcomes were organized by the level of injury for children (e.g., the severity of injuries and the frequency of injuries). For subjective traffic safety (perception of safety), the outcomes were organized by the level of perceived safety (e.g., unsafe, traffic danger, or high risk to walk or bike). The results are summarized by using one term, children’s traffic/road safety to highlight the links with the traffic and built environment variables including infrastructure and road design features that had a relationship with traffic safety for children using active transportation.

Based on the final relevant articles, to compare the objective and perceived safety results, we organized the results into one table that contains the variables of influence. The results of studies that pertain to each variable are described as unsafe/dangerous, no correlation, and safe/less dangerous. For results that examined a statistical relationship, the words unsafe or dangerous pertain to built environment variables that positively related to injuries among children (25 articles) or a perception of being unsafe (5 articles).

For perceived traffic safety, there were 13 studies in total. Eight studies did not examine any statistical relationship with the built environment. Five studies examined where a statistical relationship with built environment exists. The first used qualitative and quantitative methods (focus group, Likert scale), while the second only considered the quantitative methods.

## 3. Results and Discussion

**Figure 2:**
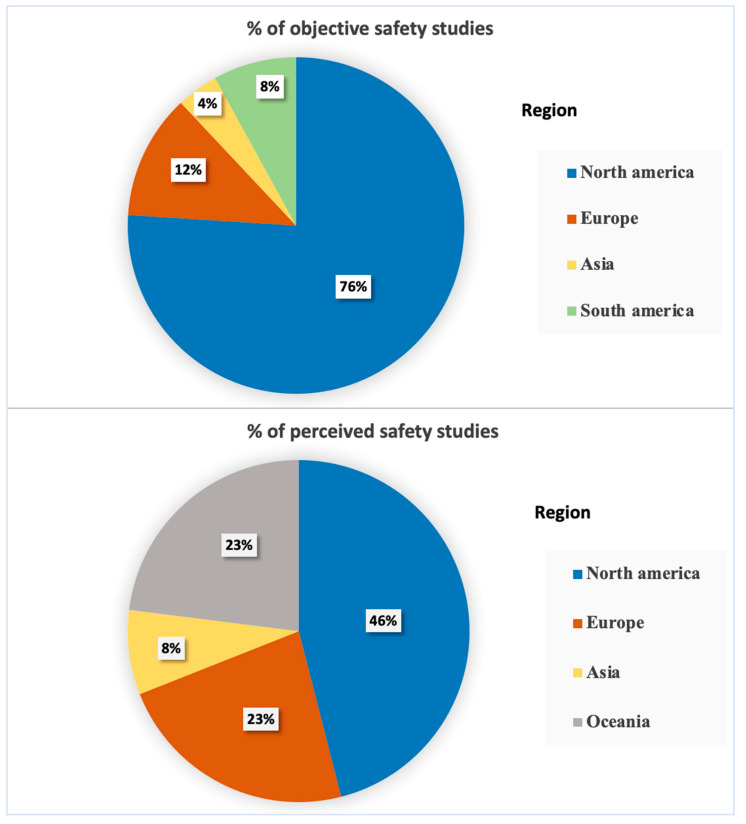
Percentage of identified studies by region.

A total of 25 articles related to child pedestrian or bicyclist collisions (whether or not they resulted in injury or death; Appendix B), and a total of 13 articles related to perception of safety (Appendix B). Of the articles retrieved, 76% of objective safety studies and 46% of perceived safety studies were from North America, representing a large majority (Figure 2).

**Figure 3:**
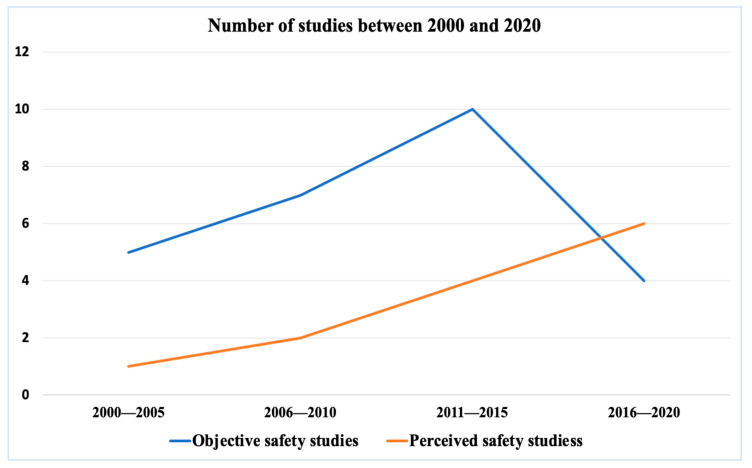
Number of objective and perceived safety studies in the period 2000–2020.

From the final selected studies, the number of perceived safety studies that examined the relationship between the built environment and perception of safety increased between 2000 and 2020 (Figure 3). However, most objective safety studies were found between 2011 and 2015, and few articles in the last five years (between 2016 and 2020).

**Figure 4:**
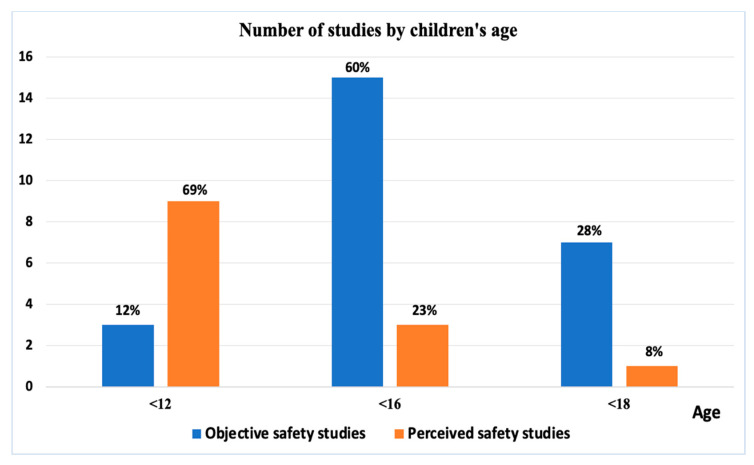
Number of objective and perceived safety studies by children’s age.

From the final selected studies, younger children were more considered in perceived safety studies that examined the relationship between the built environment and perception of safety compared to objective safety studies (Figure 4). It seems that in this age (<12) of elementary school, parents feel more concerned about traffic safety, and parents’ and children’s perception of safety tends to be very important in walking or cycling independently. Younger age groups tend to be accompanied by an adult compared to older children. Those studies focused on younger children. However, few objective safety studies (12%) examined only elementary school-aged children, and the majority of objective safety studies also included children from secondary and high school. The majority of objective safety studies included children less than 16 years old. The data of objective safety studies that examined collisions involving children come from police and hospital reports.

**Figure 5:**
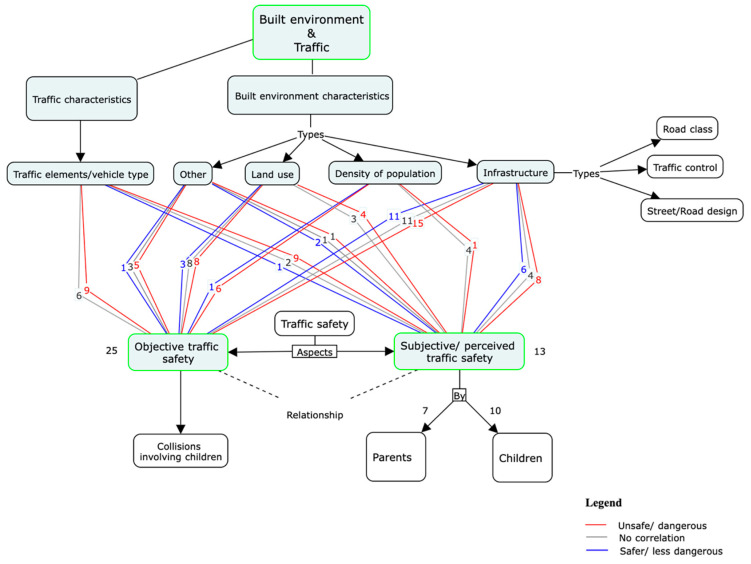
Number of studies that included built environment and traffic variables related to objective and perceived traffic safety.

The built environment variables identified in the selected studies were grouped by: infrastructure (road/street design, road type and traffic control), population density, land use type and other characteristics such as distance to school. Traffic characteristics were regrouped by traffic elements such as traffic speed and volume, and vehicle type such as a motor vehicle. Figure 5 shows the number of studies that included built environment and traffic variables related to objective and perceived traffic safety.

Table 1 shows the results of built environment characteristics related to objective and perceived traffic safety.

### 3.1. Traffic Elements and Traffic Safety for Children

The two most common traffic variables that have a negative relationship with traffic safety for children, and thus a positive relationship with collisions involving children were high traffic speed and high traffic volume.

#### 3.1.1. Speed

Increased speeds were generally associated with worse outcomes. A previous review on child pedestrian collisions also found such associations [19]. Street segments with a high speed limit increase the probability of injuries among children who travel to school [40], and increase the likelihood of injuries and fatalities for middle and high school-aged children compared to elementary school-aged children [41]. Speeds often used in cities (>45 and >50 km/h) are associated with injuries among children and collisions [39,42]. However, two studies did not find a relationship between injuries among children and speed [43,44]. No correlation was found between the risk of injury and average traffic speed (>50 km/h) at both intersections and at mid-block [43]. One study focused only on collisions likely related to school travel (weekday, between 7:00 a..m and 5:00 p.m.) and the other [44] examined only cyclists omitted to emergency rooms and compared those who were severely injured (had to stay in the hospital) with those who were not. No differentiating relationship was found for posted speeds above 30 km/h between those two groups.

#### 3.1.2. Traffic Volume

Seven studies found a positive relationship between traffic volume and collisions of children. Two studies [40,45] showed a positive relationship between traffic volume and injuries among children in two periods. However, a point of difference can be seen for the summer versus school period, with one finding increased traffic was significant for both [40], while the other found it was significant only during the summer period [45]. A positive impact of average traffic volume was found on the child pedestrian/cyclist casualty rate on classified and unclassified roads. In that study, a classified road is a main or principal road [46]. A high volume of vehicles was related to a higher risk of road traffic injuries involving child pedestrians [42]. The density of traffics increased collision risk [47], and higher rates of collisions occurred in areas with high traffic volume [48]. High traffic flow and volume may create congestion, where high traffic congestion was associated with the location of traffic collisions around residential areas [39]. However, no relationship between injuries among children and average traffic flow (per 1000 vehicles) at mid-block was found. However, there is a positive relationship between average traffic flow and injuries among children at intersections [43]. One study also found that high traffic flow (high number of arriving vehicles in area of focus) was not significantly related with objective safety [47].

Regarding perception of safety, no studies examined a statistical correlation between vehicle/traffic speed and perception of safety. Two studies examined a statistical relationship between traffic volume and parental perception of safety [49,50]. Heavy traffic was negatively correlated with parental perception of safety only for boys near school [49], while it was not significantly correlated with parents’ perception of traffic danger along school routes [50].

However, both parents’ and children’s perceptions of traffic safety were examined based on qualitative and quantitative studies. The results show that high vehicle speed and high traffic volume are the main factors that relate to unsafe perceptions for both children and parents. Parents feel that it is unsafe when children travel on roads with high vehicle speed [51,52,53]. Children also do not feel safe when walking or cycling with the presence of high-speed vehicles [53,54,55,56], except for one study [52] which found that children indicated their environment was less dangerous than parents (mothers) in the presence of high-speed vehicles and high traffic volumes. Children [53,54,56,57] and parents [51,52,58] both felt that high traffic volumes were unsafe.

#### 3.1.3. Vehicle Types

Motor vehicle collisions were associated with severe injury for child bicyclists [44]. Bicycling frequency (number of uses per time) was not statistically significant to severe injury in child bicyclists, but may decrease the likelihood of severe injuries in child bicyclists [44]. For child pedestrians, higher walking rates were not found to be associated with a higher risk of motor vehicle collisions [37]. Higher rates of walking to school were not linked to injuries, and there was no significant link between the proportion of students walking to school and vehicle–pedestrian crashes [36]. For perception of safety, parents feel that the high density of heavy vehicles could decrease the safety of children when using active transportation modes [53].

### 3.2. Built Environment Characteristics Related to Traffic Safety for Children

The relationship between built environment characteristics and objective and perceived traffic safety for children was examined based on previous studies in the field. The variables were regrouped on subsections under this built environment theme: infrastructure (including traffic control, road class, and street/road design), population density, land use, and other variables (e.g., distance and school location).

#### 3.2.1. Infrastructure

##### Traffic Control

The density of traffic lights and the presence of traffic lights (versus no traffic light) were associated with more collisions involving children [36,37]. The reason may be because traffic lights are installed at dangerous crossings. In the previous review on child pedestrian injuries, traffic control devices were found to be protective against injuries [19]. In this review, we find that a higher density of traffic lights was identified as a risk factor in the inner suburbs (close to the center of the city) and had a positive association with motor vehicle collisions [37]. However, it may also increase the number of children who walk [36]. One study examined its statistical relationship with perception of safety; it showed a positive correlation between parental perception of safety and density of traffic lights [50]. However, the absence of signals at intersections or crosswalks was perceived as safer by children [52].

The presence of stop and yield signs were related to a lower risk of collision involving child pedestrians at intersections [43]. One qualitative study found that children perceived the presence of stop signs as safe or less dangerous [56]. In another study, roads without traffic signs were one of the factors related to child pedestrian crashes [59]. On the other hand, traffic signs present at mid-block were not statistically significant with child pedestrian collisions [43]. Regarding perception of safety, one study showed that the presence of a school zone sign was positively related to a high risk of child pedestrian crashes, and increased the perceived crash risk among children at intersections [60]. In that study, child participants were instructed to indicate the locations they believed had the highest risk of collision.

Intersections with no controls presented a lower risk of child pedestrian motor vehicle collisions [43], while uncontrolled mid-block crossings were related with a high severity of injuries among children compared to signalized intersection [61]. At signalized intersections, vehicles are obliged to stop in front of a red light, while at uncontrolled mid-block, it may be that drivers were not obligated to stop vehicles such as at traffic lights, or that the driver population is not well trained, or that the street design does not help them stop. Regarding perception of safety, only one study examined a correlation between mid-block crossings and perception of safety [50]. The result of this study showed that dangerous mid-block crossings were related to higher perceived route danger. It seems that uncontrolled mid-blocks are not safe places to cross compared to intersections.

Traffic calming

The results of traffic calming for objective studies were mixed. Traffic calming is intended to control traffic, generally with the intention to improve safety. In the previous review on child pedestrian injuries [19], traffic calming was found to improve safety (reduce the incidence or severity of collisions). In two of the five studies in this review, a positive relationship was found between traffic calming and collisions [36,37]. The finding that more traffic calming measures were positively associated with higher collision rates may be surprising. However, it is possible that traffic calming was installed in areas with high collision rates and a high concentration of injuries. For one of the two studies [37], no relationship was found when considering all locations, but a positive relationship with child pedestrian–motor vehicle collisions was observed for households in inner suburbs (not for those in the downtown core). In contrast to the above, three studies found a negative impact of traffic calming on injuries among children [62,63,64]. Examining the effect of traffic calming in deprived areas, traffic calming was related to higher reductions in injuries and there is a significant relationship between density of traffic calming and a reduction in child pedestrian injuries [62]. Traffic calming with speed bumps was found to reduce the occurrence of collisions with children [63,64]. Speed bumps were related to a lower risk of injuries among children in their neighborhood and in front of their home [63], and the decrease in the number of pedestrian motor vehicle collisions was larger for children than for adults [64].

A total of three studies did not find a correlation between objective traffic calming and parental perception of safety [49,50,60]. One qualitative study [55] showed that children perceived traffic calming as safe or less dangerous.

Crossing guards

Several studies [36,37,65] identified that the presence of a school crossing guard was associated with higher motor vehicle collisions involving child pedestrian. This is consistent with the previous review [19]. In agreement with that review, we suggest that school crossing guards may be put in place in dangerous crossings or intersections with high collision risk, which may explain the positive relationship between the presence of school crossing guards and injuries among children in those studies. In somewhat contrast, two studies found no statistical relationship. One study [43] found that the presence of school crossing guards was not statistically significant and that there is no relationship with child pedestrian safety at intersections in general, while the other examined schools in residential areas [39].

Regarding perception of safety results for crossing guards, two studies of perception of safety showed that crossing guards increased the perception of safety of children. The presence of crossing guard was related to lower perceived danger by parents along school routes [50]. One qualitative study [56] indicated that children feel safer when crossing guards are present. As such, there is possibly a conflict between the perceived safety and likelihood of collisions. Again, it may be that crossing guards are found at more dangerous intersections.

##### Road Class

Road class for motor vehicle

Main roads, including arterial and collector roads, were found to be related to injuries among children in several studies [38,40,64,66]. This is consistent with the previous review on child pedestrian collisions [19]. Arterial roads, compared to local roads, may increase the probability of school-aged child pedestrian crashes near schools [38,40,66]. Collector roads, compared to local roads, were also related to more motor vehicle collisions involving child pedestrians [64]. Arterial roads may have a higher speed limit compared to local roads, which may influence the risk of collision. Collector roads may be dangerous because they often transfer traffic (higher traffic volume than local streets) from local streets to arterial roads. In contrast, there is no association between the risk of collision involving child pedestrians around schools and the density of arterials (arterials per area) [36,65]. Highways or freeways were found to increase the probability of collision risk in one study [40], though they were not associated with injuries among children in another [39]. Local roads decreased the likelihood of collisions [47], and they were associated with a lower risk of collision [38,40]. However, one study found that schools located on local roadways were found to experience more collisions than other locations [67].

Regarding perception of safety results, only one study examined the correlation between road type and perception of safety [50]. In this study, collector roads were found to be associated with parents’ perception of low danger along school routes compared to arterial roads [50].

Road class for active transport

Sidewalks are designated places to walk, though their relationship with safety is not always clear. The previous study on child pedestrians [19] found that they were associated with an increase in injury, though those authors point out that there may be more child pedestrians along such routes. In this review, sidewalks were related to fewer crashes involving children compared to roads without sidewalks around the school [40]. Streets with a high proportion of missing sidewalks were found to increase the probability of school-aged child pedestrian crashes [38]. Sidewalks and bike lanes are designated active travel infrastructure. However, in studies [36,40,43], sidewalks and bike lanes were not statistically significantly related to injuries among children. Crosswalk density could increase the probability of child pedestrian crashes near schools [38], though it was not correlated with injuries among children around neighborhood environment [39]. Infrastructure with pedestrian bridges was related to fewer collisions [39], though they can be significant barriers to people with mobility problems such as parents with strollers and people with physical disabilities.

Regarding perception of safety results, three studies examined the correlation between perception of safety and active transportation roads [50,57,60]. The presence of sidewalks was not statistically related to perceived traffic danger by children at intersections [60]. Density of missing sidewalks was not statistically related to perceived danger along school routes by parents in Toronto, Canada [50]. In contrast, children feel that sidewalks are a safer place to walk [56,57,68]. However, the presence of crosswalks was positively related to children’s perception of crash risk [60]. The presence of pedestrian infrastructure was positively related to perception of a safe walk to school among adolescents [57]. Separate bicycle lanes and walking paths from roads were perceived safer for parents and children [53,68].

##### Street/Road Design

One-way streets

One-way streets were associated with higher collision rates [37]. One-way streets were positively associated with more collisions, though they were also positively associated with walking to school [36]. As such, they may increase walking rates, but also increase collision risk, which would be a bad combination if found to be a consistent finding. This may be because there are no conflicting movements and thus people drive at higher speeds or when arriving at an intersection with a one-way street they do not pay much attention to both directions of the road [69]. However, a different study found that one-way streets were not associated with injuries among children at intersections and mid-block [43].

One study examined the relationship between one-way streets and perception of safety. The result of that study showed that one-way streets were not associated with parents’ perception of safety along school routes [50]. In contrast, a separate study found that parents feel that it is unsafe for their children to cycle in one-way streets [53].

Street width

A street width under five meters (<5 m) or between five and eight meters was statistically significant and positively associated with traffic collisions involving children compared to wide streets (>15 m) in Iran [39]. In contrast, both parents and children in the US feel safe walking and cycling in narrow streets [68].

Absent lane demarcations were related to higher injury rates among children, and roads without lane demarcations may create more chaos on the way and contribute to uncontrolled traffic flow [42].

Divided versus undivided roads

The likelihood of a crash decreases on undivided roads as the number of lanes increases, whereas it increases on divided roads [41]. This may be explained several influences. Drivers may speed more when the number of lanes increases on divided roads. Second, drivers may pay more attention when there is no median, which could reduce the likelihood of crash occurrence [41]. Another explanation is that multiple-lane roads without a median are simply too dangerous that people do not attempt to across. Wider road width was perceived to be positively associated with crash risk among school-aged children [60].

Intersection density

An increase in the length of the road was related to higher risk of collision involving child pedestrians [43]. Longer roads (direct road without intersections) may increase the possible contact between pedestrian and vehicle. Straight roads are associated with high-risk locations for traffic safety for children. Straight roads in this study were situated in areas with high traffic flow and speed which also increase the risk of injuries among children [59].

High street connectivity with higher intersection density, average block length and connected node ratio appears to be a factor related with a low risk of child pedestrian and cyclist injuries compared to low street connectivity [70], and it was measured in a 5 km buffer around school. It may increase safe active transportation among children as areas with high street connectivity offer more route choices and children may be able to avoid dangerous streets. However, intersection density was found to not be statistically significant for collisions involving children for several studies [36,38,40,71]. Further, intersection density was negatively associated with perception of safety [49,57,72], and it related to more unsafe crossing places for children.

Bus stop density

Bus stop density was not associated with child pedestrian crashes across school neighborhoods [38] and at mid-block crossing [43]. Streets with a higher density of transit stops increase crash risk for 100 feet buffers of each street segment around a school [40]. Transit access, which was defined as the percentage of households in an area which are less than 0.5 miles from a transit stop, was not related to traffic safety children, but it may decrease the crash risk of other pedestrian age groups [73].

Dead-end roads

The density of dead-end roads was not associated with injuries among children [37]. For perception, the results are contradictory. One study showed that dead-end roads were positively related to parental perception of safety along school routes [50]. In a different study, children and parents felt that routes with a high density of dead-end roads are dangerous [68].

Road density

Road and network density were not associated with objective measures of safety [65,66,73]. Regarding perception of safety, road and network density were not correlated with perceived traffic safety [60].

#### 3.2.2. Population Density

High multifamily dwelling density decreased the likelihood of child pedestrian collisions [36]. For perception of safety, high multifamily dwelling density is not related to perceived crash risk [50].

Several studies found a negative relationship between population density and injuries among children [38,45,65,67,73], though it was also found to be related to risk of exposure in areas near public school [73]. This is in contrast to the previous review on child pedestrian injuries [19] though it is not clear which articles they base this finding on. High population density may increase walking proportions in areas around elementary schools, though such areas were found to be linked to high-risk exposure, and the high population density could be related to more trips for children to school. Youth population density was negatively associated with safety of children and increased injury rates during the school year [45]. Additionally, a study [65] found that population density and residential density were related to child pedestrian risk around schools.

For perception of safety, population density, including residential density, was not associated with perceived safety in several studies [50,57,60]. However, one study [72] showed a negative relationship between residential density and perception of safety.

#### 3.2.3. Land Use

Many studies show that commercial land use was not related to either objective traffic safety [36,37,40] or perceived traffic safety [50,60]. However, commercial land uses may generate more interactions between motor vehicles and pedestrians and increase the number of crashes within a 100 feet buffer along each street segment [40], and injuries near school [38]. One study indicated that commercial access was related to a high severity of crashes within school neighborhoods involving adults because of high pedestrian demand, but it was not significant for children [73]. In Toronto, Canada retail density was not related to perception of safety [49]. High street vendor density increased the risk of injuries for child pedestrians in Lima, Peru [42], though this may also be related to such activities occupying pedestrian infrastructure.

Arterial roads were more often associated with commercial land uses, while residential land uses were more often associated with local roads which are generally more disconnected from traffic [40].

For residential land use, studies [37,40,71] showed a negative relationship with injuries among children, while studies [38,39,40] indicated that there is no correlation with objective or perceived traffic safety [60]. In a separate study [37], areas with high residential land use had a protective influence and may be a safe place for children. Residential land use was associated with low speed limits and traffic flow [37]. Areas with high proportions of residential land use were found to be safer for child pedestrians, maybe because more traffic calming was located in high-density residential areas [71]. Finally, one study [39] found that residential areas were not significantly associated with traffic collisions.

The effect of mixed and diverse land use showed a positive relationship with injuries among children [43,64,65], while other studies [38,73], [44] indicated that there is no correlation. The previous review on child pedestrian injuries [19] suggested a positive relationship. For perception of safety, mixed land use was found to be positively related to unsafe walking and cycling to school among adolescents in area within 500 m of school location [57]. In a similar study [72], mixed land use was not related to unsafe walking and cycling to school among adolescents [72].

A positive relationship between mixed land use and motor vehicle collisions involving children was found after speed bump installation [64]. Mixed land use was defined as the distribution of all land use types such as residential, commercial, institutional, industrial, and other land use types. One study in Montreal found the same result, that the diversity of land use was positively associated with higher crash risk around schools [65]. A study [43] examined the effect of mixed land use and non-residential land use on traffic safety children at intersections and mid-block crossings. They found a negative effect of mixed land use on traffic safety children at intersections, but it was not significant at mid-block crossings. Mixed land use may contain various types of land uses including commercial centers, which may generate more interaction and complex conflicts between vehicles and pedestrians at intersections compared to mid-block [43]. The type and severity of pedestrian injuries in children may be related to land use variables. One study [71] showed that secondary retail could be an issue for children’s active transportation safety. The educational sites including schools, libraries, and universities were related only to killed or serious injury. Primary retail such as shopping centers was related to slight injuries on the weekend.

##### Near Schools

Areas near schools were associated with more crashes, especially for middle and high school children. This was explained as the areas near middle and high schools being associated with high speed and multilane roadways compared to areas near elementary school [41]. Additionally, zones near or with schools were related with risk of injuries [39,74]. In contrast, [43] found that areas near schools (within 150 m of school) were not related to injuries among children. That study investigated child pedestrian collisions at intersection and mid-block locations.

##### Near Parks

Living near parks was related to high child pedestrian fatalities compared to living near a school in a study of six cities in the US [74]. This may be due to the existence of unsafe streets next to the parks. The authors of that study also suggest that it might be a lack of awareness that parks are associated with a high concentration of collisions in the US.

##### Public Parking

The existence of public parking was found to be statistically significant with traffic collisions in Iran [39], while two studies in North America found that there is no relationship between parking and traffic safety for children. Off-street parking lots were found to not be statistically significant for child pedestrian and all pedestrian ages [73]. On-street parking was not statistically related to motor vehicle collisions involving children near the mid-block location [43]. Regarding perception of safety results, one study [50] indicated that the existence of double parking along school routes was not related to parental perceived danger. Children [54,56] and parents [51] feel that the presence of street parking along school routes decreases safety for children.

##### Other Land Use

Office, industrial and park land use were not related to motor vehicle collisions involving child pedestrians in many studies [36,37,38,40,73].

#### 3.2.4. Other

Distance can be related to the amount of exposure to danger. The previous review [19] on child pedestrian injuries found that an increase in distance increased injury incidence or severity. In our review, a study [39] showed that closer distances <100 m had fewer injuries among children than farther distances. The distance between school and intersection or mid-block was not related with traffic safety for children. One study showed that a longer distance to/from school was negatively related to parental perception of safety for children [49].

The rest of the results showed that lighting, weather, weekday peak time, cycling destination, and traveling or crossing with companions were all not associated with injuries among children.

**Figure 6:**
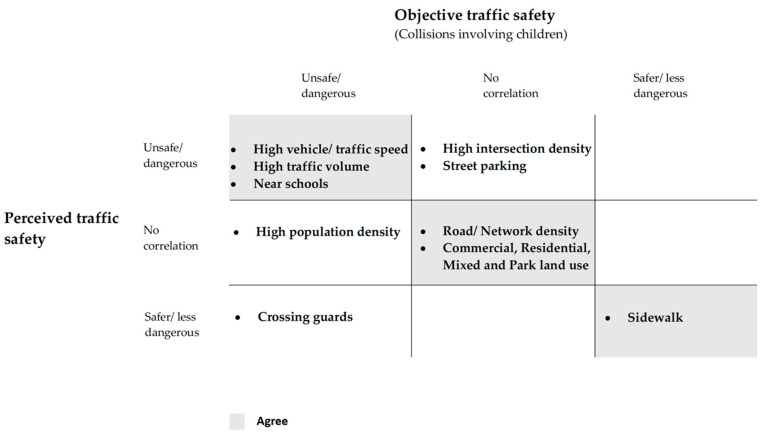
Main results for objective and perceived traffic danger (agree/disagree).

Figure 6 summarizes the main findings of this study. The main results were regrouped by the level of agreement between objective and perceived traffic safety.

Quite a number of measures were not sufficiently studied to have a clear influence on either objective or subjective traffic safety. A number of objective measures had no (or only 1) similar measure in subjective studies, such as road hierarchy (local, collector, arterial), uncontrolled intersections, block length, and retail type.

In a few cases, objective studies were not found by our review related to subjective measures such as having cross major roads, presence separated bike/pedestrian paths, and walkability index.

In general, most objective research is from a North American context that may not represent many other countries due to its high car ownership, car-centric development (resulting in large straight roads and ample parking), and quite strict land use segregation.

**Table 1:** Statistical relationships between built environment related to objective and perceived traffic safety for children.

Variables	Objective Traffic Safety (Collisions or Injuries)			Perceived Traffic Safety (i.e., Not Safe)		
	Safer/Less Dangerous	No Correlation	Unsafe/Dangerous	Safer/Less Dangerous	No Correlation	Unsafe/Dangerous
1. Traffic						
Traffic elements						
High vehicle/traffic speed		[43] ^e,f^, [44]	[39,42], [40] ^b^*, [41] ^n^	[52] ^2,(c)^		[51] ^1,(p)^, [52] ^2,(p)^, [53] ^1,(p,c)^, [54] ^2,(c)^, [55] ^1,(c)^, [56] ^1,(c)^
High traffic volume/flow/Too much traffic		[43] ^f^, [45] ^s^, [47]	[39,40,42,45,46,48], [43] ^e^	[52] ^2,(c)^	[49] (*) (girls), [50] (*)	[49] (*) (boys), [51] ^1,(p)^, [52] ^2,(p)^, [53] ^1,(c)^, [54] ^2,(c)^, [56] ^1,(c)^, [57] ^2,(c)^, [58] ^2,(p)^
Vehicle types						
Impact with motor vehicle			[44]			
Heavy vehicles						[53] ^1,(p)^
Bicycling frequency		[44]				
Walking proportion (more walking)		[36,37]				
2. Built environment						
2.1. Infrastructure						
2.1.1. Traffic control						
Higher density of traffic lights		[37]	[36] ^j^, [37] ^j^	[50] (*)	[52] ^2,(p)^	[52] ^2,(c)^
Presence of traffic/stop signs	[43] ^e^, [59]	[43] ^f^		[56] ^1,(c)^		[60] ^e^ (*)
Uncontrolled intersection vs. controlled	[43] ^e^					
Higher density of flashing beacon						[50] (*)
Dangerous or uncontrolled mid-block locations			[61]			[50] (*)
Traffic calming	[62,63,64]	[37]	[36], [37] ^j^	[55] ^1,(c)^	[49] (*), [50] (*), [60] ^e^ (*)	
Crossing guard presence		[39], [43] ^e^	[36,37], [65]	[50] (*), [56] ^1,(c)^		
2.1.2. Road class						
Road for motor vehicle						
Main roads (arterial/collector roads) vs. local roads		[36,65]	[38,40,64,66]			
Collector roads vs. arterial roads				[50] (*)		
Local roads	[38,47], [40] ^b^*		[67]			
Highways or freeways		[38,39], [40] ^b^**	[40] ^b^*			
Driveways		[73]				
Active transport infrastructure						
Sidewalk	[38], [40] ^b^*	[36], [40] ^b^**, [43] ^f^		[56] ^1,(c)^, [57] ^2,(c)^, [68] ^2^	[50] (*), [60] ^e^ (*)	
Crosswalk		[39]	[38]		[50] (*), [52] ^2,(p)^	[52] ^2,(c)^, [60] ^e^ (*)
Bicycle lane		[40,43] ^f^		[57] ^2,(c)^		
Separate bicycle lane and walking path				[53] ^1,(p)^, [68] ^2,(p,c)^		
Presence of pedestrian bridge and infrastructure (e.g., refuge island)			[39]	[57] (*)		
2.1.3. Street/Road design						
One-way street		[43] ^e,f^	[36,37]		[50] (*)	[53] ^1,(p)^
Narrow streets			[39]	[68] ^2,(p,c)^		
Absence of lane demarcations			[42]			
Larger road width	[39]		[41] ^n^			[60] ^e^(*)
Total road length (longer)			[43] ^f^			
Longer block length			[38]			
Straight road sections			[59]			
Intersection place		[37]	[59]			
Presence of major road crossings					[49] (*) (boys)	[49] (*) (girls)
Density of transit stops		[38,73], [40] ^b^**, [43] ^f^	[40] ^b^*			
Dead-end roads/No-cul-de-sacs		[37]		[50] (*)		[68] ^2,(p,c)^
Road/Network density		[65,66,73]			[60] ^e^ (*)	
Intersection/junction density		[36,38,71], [40] ^b^**				[49] (*), [57] (*), [72] (*)
2.2. Population density						
High street vendor/retail density			[42]		[49] (*)	
High multifamily dwelling density	[36]				[50] (*)	
Population density			[38,45,65,67,73]		[60] ^e^ (*), [50] (*), [57] (*)	[72] (*)
2.3. Land use						
Land use type						
Walkability index						[57] (*)
Commercial land use		[36,37], [40] ^b^**	[40] ^b^*, [38]		[50] (*), [60] ^e^ (*)	
Commercial access		[73]				
Residential land use	[37,71], [40] ^b^*	[38,39], [40] ^b^**			[60] ^e^ (*)	
Mixed, diversity or non-residential land use		[38,73], [43] ^f^	[43] ^e^, [64,65]		[72] (*)	[57] (*)
Secondary retail			[71]			
Primary retail		[71]				
Educational sites		[71]				
Zone near school (School present)		[43] ^e,f^	[39,74]			[54] ^2,(c)^
Living near park			[74]			
Street parking		[43] ^f^, [73]	[39]		[50] (*)	[56] ^1,(c)^, [54] ^2,(c)^, [51] ^1,(p)^
Other land use						
Office land use		[38,40]				
Industrial land use		[38,40]				
Park land use		[36,37,38,40,73]			[60] ^e^ (*)	
2.4. Other						
Distance to/from school	[39]		[40]			[49] (*)
Lighting (lack or no lighting)		[44]		[52] ^2,(c)^	[52] ^2,(p)^	
Older-amalgamated city vs. inner suburbs			[64]			
Traveling or crossing with companions		[44]		[56] ^1,(c)^		
Weather		[44]				
Weekday peak time		[44]				
Cycling destination (school, work, shopping, other)		[44]				
Elementary school (location)		[45]	[73]			
Middle school location		[45,73]	[45] ^s^			
High school location		[45]				
Child pedestrian activity			[43] ^e,f^			

(*) Statistical relationship with objective built environment; ^(p)^ parents’ perception; ^(c)^ children’s perception. ^1^ Qualitative (e.g., focus group/discussion); ^2^ quantitative (e.g., Likert scale/ratio or %); ^s^ during school period; ^b^ within/near school zone; ^b^* (<100 feet buffer); ^b^** (<half mile buffer); ^e^ at or near intersection; ^j^ inner suburbs; ^f^ at or near mid-block; ^n^ older children vs. younger.

## 4. Conclusions

This systematic review examined the relationship between objective and perceived traffic safety for children. Parents’ and children’s perception of traffic safety indicated that they feel that high vehicle speed and high traffic volume are the key dangerous factors for traffic safety children when walking or cycling.

The results of objective traffic safety for children indicated that high vehicle speed and high traffic volume were the main determinants of injuries among children. For built environment variables, sidewalk was negatively related to motor vehicle collisions involving children. high density of traffic lights and roads without signs also contributed to injuries according to some studies. In comparison to intersections with traffic lights, those with yield signs, stop signs, and even no intersection control were associated with greater traffic safety for children. Arterials and collector roads are associated with more injuries, while local roads increase the safety of children. Intersection density and road or network density were not related to injuries among children in several studies. For land use characteristics, higher residential density was related to fewer injuries among children in some studies and high multifamily dwelling density was positively associated with traffic safety for children in one study.

The main results for perception of safety showed that sidewalk was related to perception of safety. Intersection and junction density were related to perceptions of being less safe. Traffic calming, street parking, commercial and residential land use were not found to be statistically associated with perceived safety.

Comparing results between objective and perceived traffic safety showed that only sidewalk was related to perception of safety and lower risk of collisions involving children. The presence of a crossing guard increased perception of safety but was positively related to collisions involving children. Intersection density was related to unsafe perceptions but was not statistically associated with objective traffic safety. Additionally, population density was found to be related to injuries among children, but not to perception of safety.

This study examined the association between the built environment and traffic safety for children. Many identified studies investigated the relationship between traffic collisions involving child pedestrians/cyclists, while few studies examined the link with perception of safety. Future research should shed more light on the relationship between the built environment and perception of safety as this can influence the likelihood of active and independent trips for children.

## Data Availability

Not applicable.

## Appendix A

**Table A1:** Search terms used in systematic review by database.

Databases	Strategies Used for Objective Traffic Safety	Strategies Used for Perceived Traffic Safety
Web of science (2000–2020)	AB=(child* OR school* OR infant OR Adolescent* OR youth) AND AB=(injur* OR accident* OR crash* OR collision OR death* OR casualt* OR fatal*) AND AB=(traffic OR environment* OR build OR built OR design OR socio* OR street OR road OR location OR geograph* OR gis OR area OR Neighbo* OR spatial OR urban OR intersection* OR infrastructure* OR sidewalk* OR way OR ways OR crosswalk* OR path OR paths OR pathway OR land OR speed OR signs OR densit* OR flow OR vehicle OR vehicles OR car OR cars) AND AB=(pedestrian* OR walk* OR cyclist* OR bicycling OR bicycl* OR cycling OR “active transport*” OR “active commut*” OR travel)	AB=(parent* OR mother* OR father* OR child* OR infant OR Adolescent* OR school) AND AB= (perception OR subject OR view* OR perceived OR qualitative Or subjective) AND AB=(safet* OR risk* OR securit* OR unsafe* OR danger* OR barriers) AND AB=(transport* OR traffic OR speed OR signs OR densit* OR flow OR vehicle OR vehicles OR car OR cars OR environment* OR Build* OR design OR socio* OR street* OR road* OR location OR geograph* OR Neighbourhood* OR neighborhood* OR intersection* OR infrastructure* OR sidewa* OR sidewalk* OR way OR ways OR crosswalk* OR path OR paths OR pathway OR land) AND AB=(pedestrian* OR walk* OR cyclist* OR bicycling OR bicycl* OR cycling OR “active transport*” OR “active commut*” OR travel)
PubMed and Medline (2000–2020)	(((child*[Title/Abstract] OR school*[Title/Abstract] OR infant[Title/Abstract] OR Adolescent*[Title/Abstract] OR youth[Title/Abstract]) AND (injur*[Title/Abstract] OR accident*[Title/Abstract] OR crash*[Title/Abstract] OR collision[Title/Abstract] OR death*[Title/Abstract] OR casualt*[Title/Abstract] OR fatal*[Title/Abstract]) AND (traffic[Title/Abstract] OR environment*[Title/Abstract] OR build[Title/Abstract] OR built[Title/Abstract] OR design[Title/Abstract] OR socio*[Title/Abstract] OR street[Title/Abstract] OR road[Title/Abstract] OR location[Title/Abstract] OR geograph*[Title/Abstract] OR gis[Title/Abstract] OR area[Title/Abstract] OR Neighbo*[Title/Abstract] OR spatial[Title/Abstract] OR urban[Title/Abstract] OR intersection*[Title/Abstract] OR infrastructure*[Title/Abstract] OR land[Title/Abstract] OR speed[Title/Abstract])) AND (pedestrian*[Title/Abstract] OR walk*[Title/Abstract] OR cyclist*[Title/Abstract] OR bicycling[Title/Abstract] OR bicycl*[Title/Abstract] OR cycling[Title/Abstract] OR “active transport*”[Title/Abstract] OR “active commut*”[Title/Abstract] OR travel[Title/Abstract]) AND ((“2000/01/01”[Date-Publication]: “2020/12/31”[Date-Publication])	(((“safety”[Title/Abstract] OR risk[Title/Abstract] OR security* [Title/Abstract] OR unsafe[Title/Abstract] OR danger[Title/Abstract]) AND (Traffic [MeSH Major Topic] OR environment [MeSH Major Topic] OR Build [MeSH Major Topic] OR Built [MeSH Major Topic] OR design [MeSH Major Topic] OR socio [MeSH Major Topic] OR street [MeSH Major Topic] OR road [MeSH Major Topic] OR location [MeSH Major Topic] OR geograph [MeSH Major Topic] OR Neighbourhood [MeSH Major Topic] OR neighborhood [MeSH Major Topic] OR intersection [MeSH Major Topic] OR infrastructure [MeSH Major Topic] OR sidewalk [MeSH Major Topic] OR way [MeSH Major Topic] OR ways [MeSH Major Topic] OR crosswalk [MeSH Major Topic] OR path [MeSH Major Topic] OR paths [MeSH Major Topic] OR pathway [MeSH Major Topic] OR land[MeSH Major Topic]) AND pedestrian [MeSH Major Topic] OR walk [MeSH Major Topic] OR cyclist [MeSH Major Topic] OR bicycling [MeSH Major Topic] OR bicycl [MeSH Major Topic] OR cycling [MeSH Major Topic] OR “active transport” [MeSH Major Topic] OR “active commut” [MeSH Major Topic] OR travel [MeSH Major Topic]) AND (parent[Title/Abstract] OR mother[Title/Abstract] OR father[Title/Abstract] OR infant[Title/Abstract] OR child[Title/Abstract] OR adolescent[Title/Abstract] OR school[Title/Abstract])) AND ((“2000/01/01”[Date-Publication]: “2020/12/31”[Date-Publication]))
ScienceDirect (2000–2020)	(child OR school) AND (injur OR crash OR collision OR accident) AND (traffic OR environment OR geographic)	(parent OR child) AND (perception OR perceived) AND (traffic OR environment OR geographic)
ProQuest Dissertations & Theses Global (2000–2020)	TI (child* OR school OR Adolescent*) AND AB (injur* OR accident* OR crash* OR collision OR death* OR casualt* OR fatal*) AND ab(traffic OR environment OR Build* OR design OR socio* OR street* OR road* OR location OR geograph* OR Neighbourhood* OR neighborhood* OR intersection* OR infrastructure*) AND ab(pedestrian* OR walk* OR cyclist* OR bicycling OR bicycl* OR cycling OR “active transport” OR “active transportation” OR “active transporters” OR “active commut*” OR travel)	AB (parent* OR child* OR Adolescent* OR school) AND AB (perception OR subject OR view* OR perceived OR qualitative) AND AB(safet* OR risk* OR securit* OR unsafe* OR danger*) AND ab(traffic OR environment OR Build* OR design OR socio* OR street* OR road* OR location OR geograph* OR Neighbourhood* OR neighborhood* OR intersection* OR infrastructure*) AND ab(pedestrian* OR walk* OR cyclist* OR bicycling OR bicycl* OR cycling OR “active transport” OR “active transportation” OR “active transporters” OR “active commut*” OR travel)
Compendex (2000–2020)	((parent* OR mother* OR father* OR child* OR infant OR Adolescent* OR school) wn KY AND (injur* OR accident* OR crash* OR collision OR death* OR casualt* OR fatal*)wn KY AND (traffic OR speed OR signs OR densit* OR flow OR vehicle OR vehicles OR car OR cars OR environment* OR build* OR built* OR design OR socio* OR street* OR road* OR location OR geograph* OR neighbourhood* OR neighborhood* OR intersection* OR infrastructure* OR sidewa* OR sidewalk* OR way OR ways OR crosswalk* OR path OR paths OR pathway OR land) wn KY AND(pedestrian* OR walk* OR cyclist* OR bicycling OR bicycl* OR cycling OR “active transport*” OR “active commut*” OR travel) wn KY)	((parent* OR mother* OR father* OR child* OR infant OR Adolescent* OR school) wn KY AND (perception OR subject OR view* OR perceived OR qualitative Or subjective)wn KY AND(safet* OR risk* OR securit* OR unsafe*)wn KY AND (traffic OR speed OR signs OR densit* OR flow OR vehicle OR vehicles OR car OR cars OR environment* OR build* OR built* OR design OR socio* OR street* OR road* OR location OR geograph* OR neighbourhood* OR neighborhood* OR intersection* OR infrastructure* OR sidewa* OR sidewalk* OR way OR ways OR crosswalk* OR path OR paths OR pathway OR land) wn KY AND(pedestrian* OR walk* OR cyclist* OR bicycling OR bicycl* OR cycling OR “active transport*” OR “active commut*” OR travel) wn KY)

The asterisk * was used to include in the search variations on a root word; it allows any characters or letters that might be in its place (e.g., child* includes terms as: child, children, childhood).

## Appendix B

**Table A2:** Description of studies on objective measures of child pedestrian and cyclist collisions or injuries.

Study	Location	Outcome	Pedestrian/Cyclist	Subject/Participants (Number of Collisions/Injuries)	Data Sources	Year of Data	GIS	Study Design	Statistic Description
Abdel-Aty, M., S.S. Chundi, and C. Lee, 2007 [41]	Florida, USA	Crash frequency	Pedestrians/Bicyclists	Age: 4–18 years; number of injuries among children = 451 School level: elementary (4–11), middle (12–14), high school (15–18) children; number of schools = 157	Police crash reports	1999–2003	✔	Cross-sectional	Log-linear models (*p*-value < 0.05)
Bennet, S.A. and N. Yiannakoulia, 2015 [43]	Hamilton, Ontario, Canada	Crash frequency (minor collisions were not included)	Pedestrians	Age: 5–14 years case = 107 mid-block injuries; 92 intersection injuries School level: elementary public school	Police report	2002–2011	✔	Case–control study	Conditional logistic regression, using odds ratio, *p* was significant at 0.05 for intersection model, and 0.01 for mid-block model)
Blazquez, C.A. and M.S. Celis, 2013 [59]	Santiago, Chile	Crash frequency	Pedestrians	Age: 5–18 years School level: elementary, secondary, high school	Police officers fill out a paper	2000–2008	✔	Cross-sectional	Moran’s I index test, *p* < 0.005
Clifton, K.J. and K. Kreamer-Fults, 2007 [73]	Baltimore City, Maryland, USA	Crash frequency and severity	Pedestrians	Age: <5 and 5–15 years School level: 116 elementary, 23 middle, and 24 high school	Police reports	2000–2002	✔	Cross-sectional	Statistically significant at the 10% confidence level
Cloutier, M. et al., 2007 [66]	Montréal, Canada	Crash frequency (number of collisions)	Pedestrians	Age: 5–14 years School level: elementary school Number of schools: 331	Police reports	1995–1999	✔	Cross-sectional	Multivariate regression (*p* value) *p* < 0.05
Cloutier, M.-S. and P. Apparicio, 2008 [65]	Montreal, Canada	Risk of collision	Pedestrians	Age: 5–14 years School level: elementary public-school environment	Police report	1999–2003	✔	Cross-sectional, ecological	Poisson géographiquement pondérée (GWR)
Dissanayake, D., J. Aryaija, and D.P. Wedagama, 2009 [71]	Newcastle city, UK	Crash severity: slight, serious and fatal events; KSI: killed or serious injuries	Pedestrians	Age: <16 years	Police Force area	2000–2005	✔	Case study, ecological study	Poisson, negative binomial, bernoulli Methods, significant at 95% level of confidence
Donroe, J. et al., 2008 [42]	Lima, Peru	Injuries, risk of child pedestrian RTIs road traffic injuries	Pedestrians	Age: <18 years Final participants: (5061 households and 10,210 children; Injuries among children: case = 100, controls = 200 Environments: 40 case and 80 control School level: elementary, middle, and high school	Completed surveys	2000–2005		Cross-sectional, case control study	Logistic regression models, after adjustment (multivariate, combination of personal and environmental risk factors), 95% CI
Ferenchak, N.N. and W.E. Marshall, 2017 [74]	6 American cities, USA	Crash frequency (fatalities concentrations)	Pedestrians	Age: <18 years, number of schools with child pedestrian injuries = 332 schools School level: elementary, middle, and high school	2015 open data	1982–2012		Ecological study	Significant at 95% CIs (% differences) (schools or parks vs. neither schools nor parks)
Hagel, B.E. et al., 2015 [44]	Alberta, Canada	Crash severity (severe injury)	Cyclists	Age: <18 years; total participants = 1470, boys (72,58%), females (27,42%); cases = 119 (8.1%), controls = 1351 (91.9%), total case and controls = 1470 School level: elementary, middle, and high school	Hospital medical charts, and face-to-face, and telephone interviews	May 2008 and October 2010		Case–control study	Logistic regression models (with multiple imputation) at 95% confidence intervals (CIs), and odds ratios
Hwang, J. et al., 2017 [38]	Austin, Texas, USA	Crash frequency (probability of injury)	Pedestrians	Age: ≤18 years number of injuries among children = 130	Department of transportation	2010–2014	✔	Cross-sectional	Logistic regression analysis (*p* value), *p* < 0.05
Jamshidi, E., A. Moradi, and R. Majdzadeh, 2017 [39]	Tehran, Iran	Crash frequency (Injury)	Pedestrians	Age: 5–15 years, 64.3% boys and 35.7% girls; cases = 280, control = 560, total number = 840	Hospital supervision and surveillance	2013		Case–control study	Conditional logistic regression model, 95% CI OR, *p*-value < 0.05
Jones, S.J. et al., 2005 [62]	2 cities (A and B) from UK (not specified)	Injuries and fatalities (inequity of injuries among children)	Pedestrians	Age: 4–16 number of injuries among children = 1560	Police data	1992–2000		Time series, ecological	Using 95% confidence intervals
LaScala, E.A., P.J. Gruenewald, and F.W. Johnson, 2004 [45]	California, USA	Crash frequency (annual numbers of injuries)	Pedestrians and cyclists	Age: <16 years. Number of collisions = 717 School level: elementary schools (grades 1–5), middle schools (grades 6–8), and high schools (grades 9–12),	Police database	April 1992–March 1996	✔	Ecological study	Combines the variables of socio demographics and environment using a separate *t*-test (*p* ≤ 0.05)
McArthur, A. et al., 2014 [67]	Michigan, USA	Crash frequency (probability of crash)	Pedestrian and Bicycle	Age: 5–14 years number of child pedestrians and bicycle crashes = 7781 crashes	Police databases	2007–2011	✔	Cross-sectional	Random-effects negative binomial (*p* value) *p* < 0.05
Mecredy, G., I. Janssen, and W. Pickett, 2012 [70]	Canada	Crash frequency (Occurrence of injuries)	Pedestrians and cyclists	Age: 6–15 years; final number of students = 9021 School level: elementary, middle and high school; number of schools = 180	Hospital information, and cross-national survey (questionnaire distributed to children in classroom)	2006	✔	Cross-sectional study (national study)	Multilevel logistic regression analysis, significant at *p* < 0.01
Petch, R. and R. Henson, 2000 [46]	Salford city from United Kingdom	Crash frequency	Pedestrians and Cyclists	Age: <15 years number of casualties = 556 children	Police and Hospital	1 May 1995–31 April 1998	✔	Cross-sectional, ecological study	Multiple regression, at the 90% confidence level
Rothman, L. et al., 2012 [61]	Toronto, Canada	Crash severity (severe injury)	Pedestrians	Age: 0–17 years number of child pedestrian collision = 1394 School level: primary, secondary, high school	Police report	1 January 2000–December 2009	✔	Cross-sectional	Binary and multinomial logistic regression models, ORs of injury severity with 95% CI, significant at *p* < 0.05 level
Rothman, L. et al., 2014 [36]	Toronto Canada	Crash severity (including minimal, minor, major, and fatal injuries)	Pedestrians	Age: 4–12 years number of collisions involving children = 481 School level: elementary school; number = 118 schools, 22 (19%), and another 12 schools (10%) schools	Police report	2002– 2011		Cross-sectional	Negative binomial regression, significant at 0.05
Rothman, L. et al., 2015 [64]	Toronto, Canada	Crash severity (injury severity)	Pedestrians	Age: 0–14 years	Police report	2000–2011	✔	Quasi-experimental study	Rate ratio, 95% CI
Rothman, L. et al., 2017 [37]	Toronto, Canada	Crash frequency (injuries)	Pedestrians	Age: 4–12 years; collisions involving children: case = 513, control = 88 School level: primary school; case = 50, control = 50	Police report	2000–2013		Case–control study	Multivariate logistic regression modelling (adjusted model), significant at *p* ≤ 0.2 level
Tester, J.M. et al., 2004 [63]	Oakland, USA	Injuries including fatality	Pedestrians	Age: <15 years cases = 100 children, mean age = 6.8 (SD = 3.5), Contols = 200 children; mean age = 6.6 (SD = 3.7)	Pediatric ambulance trauma, and Police Department	1995–2000		Case–control	Multivariate conditional logistic regression, significant at *p* < 0.05
Yiannakoulias, N. et al., 2002 [48]	Edmonton, Alberta, Canada	Minor injuries	Pedestrians	Age: 0–15 years; number of child injured = 258	Hospital surveillance	1995–1999	✔	Cross-sectional, ecological	Empirical bayes estimation, with incidence ratios
Yiannakoulias, N. and D.M. Scott, 2013 [47]	Toronto, Canada	Crash frequency (injuries risk)	Pedestrians	Age: 5–14 years School level: elementary and secondary school aged children; n = 140 collision area	Police reported	2001–2008	✔	Cross-sectional, ecological design	Negative binomial regression, significant at the 0.1 level
Yu, C.-Y., 2015 [40]	Austin (TX), USA	Injury (Crash risk)	Pedestrians	Age: 5–12 years School level: 78 elementary schools (2 types: community-centered schools and suburban schools)	Officer’s crash report	2008–2012	✔	Cross-sectional	Bivariate analysis coefficient (*p* value) *p* < 0.05

**Table A3:** Description of studies of the perception of traffic safety for children’s active travel.

Study	Location	Walking or/and Cycling	Outcome (of Perception of Safety)	Perception Given by	Participants	Data Source	Year of Data	Study Design
Basbas, S. et al., 2009 [54]	Municipality of Kalamaria, Thessaloniki, and Larissa, Greece	Walking and cycling	Unsafe/safe to walk and cycle	Children (students)	Age: 11–12 years (sixth grade school) School level: 9 Elementary school	Data from survey; no GIS	2001	Cross-sectional
Christie, N. et al., 2007 [51]	10 low socioeconomic areas, UK	Walking and cycling	Perceived risk of traffic injuries	Parents	Age: 10 to 14 years	Focus groups	The project started in 2004	
Guliani, A. et al., 2014 [49]	Toronto, Canada	Walking	Danger to walk	Parents (mostly mothers)	Age: 10 and 11 years (average age 10.58) (720 students, grades 5 and 6) (52% girls and 47.5% boys) School level: 16 publics school (8 inner-urban, and 8 inner-suburban)	Survey (the project BEAT)	April 2010–June 2011	Cross-sectional
Hopkins, D. and S. Mandic, 2017 [53]	Dunedin, South Island, New Zealand	Cycling	Traffic danger to cycling	Parents and children students	6 parental focus groups (total = 25 participants), 10 student focus groups (total = 54 students), 5 co-educational schools, 5 single-sex schools (3 girls’ schools, 2 boys’ schools) School level: high school	Online interview focus group discussions	June 2014–April 2015	Cross-sectional
Lee, G. et al., 2016 [6]	Ulsan, Korea	Walking	Safety concern to walk (related with crash risk)	Child (students)	Age: 10–12 (53.9% boys); 799 children School level: 8 elementary school	Perception from questionnaire was distributed in the classroom Crash data from police report for crash	July 2015	Cross-sectional
Napier, M.A. et al., 2011 [68]	University of Utah, USA	Walking	Traffic unsafe to walk	Parents and children	Age: 10–11 year (n = 193); parents (n = 177) School level: elementary school	Survey (questionnaire was distributed in classroom); GIS measures	Spring 2007	Cross-sectional
Olvera, N. et al., 2012 [52]	East End district, East side of Houston, Texas, USA	Walking and cycling	Safety concern related to walking and cycling	Children and mothers	Age: 3rd to 5th grade; 132 children (55 boys and 77 girls) average age 10 years and; 102 mothers (mean age = 36.2 ± 77.3) School level: elementary schools	Self-reported surveys	2008–2009	Cross-sectional
Pocock, T. et al., 2019 [57]	Dunedin (New Zealand)	Walking and bicycling	Concern’s (traffic danger, unsafe) to walking and bicycling	Adolescents’ (students)	Age: 15.2 ± 1.4 years; data from 471 adolescents; 56.3% female School level: secondary schools	Online survey using GIS	2014–2015	Cross-sectional
Rahman, M.L. et al., 2020 [72]	Otago, New Zealand	Walking and cycling	Safety concerns	Children	Age: 15.2 ± 1.4 years School level: 23 high schools	Online survey	2014 and 2018	
Rothman, L. et al., 2015 [50]	Toronto, Canada	Walking	Traffic danger to walk collision rates	Parents	Age: 9–11 years (grades 4–6); final sample of parents n = 733 parent surveys School level: 20 elementary (primary school) schools	Data from parents survey (a written questionnaire); no GIS	2011	Cross-sectional
Soori, H. 2000 [58]	Newcastle upon Tyne, UK	Walking and cycling	Perceived risk (safe/unsafe)	Parents and children	Age: 7 and 9 years Participants: children = 471; parents = 416 School level: nine primary school	Surveys (self-completed)		Cross-sectional
Torres, J. et al., 2020 [55]	Quebec, Canada	Walking and cycling	Safe/unsafe to walk or cycle	Children	Age: 11 to 12 years	Focus groups	2014–2015	Cross-sectional
Wilson, K. et al., 2019 [56]	Southwestern Ontario, Canada	Walking and cycling	Safe/unsafe to walk or cycle	Children	Age: 10 to 12 years Total of 158 students	Focus groups		
